# Microalgal food supplements from the perspective of Polish consumers: patterns of use, adverse events, and beneficial effects

**DOI:** 10.1007/s10811-017-1079-5

**Published:** 2017-02-15

**Authors:** Piotr Rzymski, Monika Jaśkiewicz

**Affiliations:** Department of Environmental Medicine, Poznan University of Medical Sciences, Rokietnicka 8, 60-806 Poznań, Poland

**Keywords:** Microalgal supplements, *Spirulina*, *Chlorella*, *Aphanizomenon*, Consumer attitudes, Side effects

## Abstract

Microalgal food supplements are becoming increasingly popular due to their promising biological effects and high nutritional value, evidenced in in vitro, in vivo, and human studies. Some products of this kind have, however, raised controversies concerning their safety. At the same time, not much is known about the frequency of adverse events following the use of microalgal supplements, potential factors that may influence them, and general characteristics and behaviours of the consumer group. The present study aimed to fill this gap and surveyed a group of Polish consumers of microalgal products (*n* = 150) using an online questionnaire. As found, microalgal supplements (*Spirulina*, *Chlorella*, and *Aphanizomenon*) were popular in groups representing lacto-ovo-vegetarianism and veganism and were consumed predominantly for nutritional, immune-boosting, and detoxifying purposes. Their use was rarely discussed with specialists; the Internet constituted the most important source of information regarding these supplements. The most frequently self-reported health-beneficial effects of supplementation included the following: increased immunity, higher vitality, improved hair and skin quality, and better general well-being. Diarrhoea, nausea, abdominal pain, and skin rash were among the most often reported adverse events. Pre-existing medical conditions, namely renal failure and hypothyroidism, but not Hashimoto’s thyroiditis, were associated with increased occurrence of side effects. Those individuals who had consulted specialists as to the use of supplements reported adverse events significantly less often. A strikingly high frequency of side effects and very low consumer satisfaction were reported by a group of consumers supplementing *Aphanizomenon*-based products. In summary, the present study highlights that microalgal consumers may benefit from additional warnings of potential side effects and from consulting a qualified health specialist prior to use.

## Introduction

Food supplements are increasingly popular worldwide, including those based on microalgae (Kennedy 2005; Bailey et al. 2013). Most often, such products contain biomass of cyanobacteria belonging to the genera of *Arthrospira* (sold as ‘*Spirulina*’), former “*Aphanizomenon*” (sold as ‘AFA’), and *Nostoc* or green algae representing the genera *Haematococcus*, *Dunaliella*, and *Chlorella* (sold under the same name) and are marketed for their nutritional value and potential biological activity (Pulz and Gross 2004; Raposo and de Morais 2015). In fact, they have been shown to be a rich source of proteins, fatty acids, pigments, minerals, digestive and restriction enzymes, and various vitamins (Christaki et al. 2011; Gellenbeck 2012; Buono et al. 2014; Wells et al. 2016). As a good source of iron and bioavailable forms of vitamin B_12_ (Nakano et al. 2010; De et al. 2011; Merchant et al. 2015), they are potentially of high interest to individuals on vegetarian diets which are becoming increasingly popular in different populations (Dinu et al. 2016). Moreover, the positive effect of the aforementioned microalgae and their ingredients on a lipid profile as well as immunomodulatory, antibacterial, antidiabetic, antioxidant, and antitumour activities have been demonstrated not only in in vitro and in vivo experimental models but also, partially, in randomized clinical trials (Ramamoorthy and Premakumari 1996; Yamani et al. 2009; Anwer et al. 2012, 2013; Kwak et al. 2012; Torres-Durán et al. 2012; Panahi et al. 2013; Mazokopakis et al. 2014; Ryu et al. 2014). It has been further shown that *Chlorella* may be efficiently used in adjunctive therapy for depressive disorders (Panahi et al. 2015) whereas *Spirulina* intake can significantly increase CD4 cells and reduce the viral load in human immunodeficiency virus infections (Ngo-Matip et al. 2015). Altogether, the existing data supports the use of some microalgal food supplements as nutraceuticals for a wide range of potential health-beneficial applications.

It has been, however, reported that the quality of some microalgal supplements may be doubtful due to the presence of cyanotoxins such as anatoxin-a or microcystins (Jiang et al. 2008; Rellán et al. 2009; Manali et al. 2016), elevated concentrations of toxic metals including aluminium, nickel, and lead (Al-Dhabi 2013; Rzymski et al. 2015), and inorganic arsenic species (Hedegaard et al. 2013). The former issue may result from improper culture purity and the co-occurrence of potentially toxic cyanobacteria species, e.g. *Microcystis aeruginosa* (Vichi et al. 2012), while the latter two could be a consequence of an unsuitable location of cultivation ponds (e.g. near rural, industrial, or agricultural areas) and/or the use of certain chemicals (e.g. aluminium-based flocculants) to harvest the biomass (Papazi et al. 2010; Salim et al. 2011; Rzymski et al. 2015). Further, microalgal supplements may be potentially unsuitable for some individuals, particularly those suffering from renal failure due to high protein and phosphorus content (Kay 1991) or severe autoimmune disease, as a relapse of symptoms may occur (Lee and Werth 2004).

Microalgal formulas, like any other food supplements, are considered as foodstuffs, and contrary to medical products, their registration requires neither preclinical nor clinical studies. Generally, it can be considered as definitely less strict than that for pharmaceutical drugs (DSHEA 1994; Commission Directive 2002/46/EC). Moreover, food supplements are usually self-prescribed, and there is no responsibility to report side effects associated with their use. Consequently, the occurrence of adverse events, interactions with medicinal drugs, or effects on certain disorders are less controlled for these products. As far as microalgal supplements are concerned, the only available data regarding undesirable consequences have been presented in the form of a few case reports published in peer-reviewed journals and as a summary on the side effects of *Spirulina* use prepared by The Dietary Supplements Information Expert Committee of the United States Pharmacopeial Convention in 2011 (Marles et al. 2011). All in all, there remained a need to evaluate the frequency of side effects related to the consumption of these products in different populations, preferably accompanied by some specification of the consumer group and reasons behind their choice to use microalgal food supplements.

To address these issues, the present study identified and characterized a group of consumers of microalgal food supplements in Poland and evaluated their attitudes, behaviour, and intentions towards the use of these products. Currently, Poland represents the largest and most dynamic market for food supplements in central Europe due to the size of the population and a very high level of spending (PMR 2016). Numerous products containing microalgae, predominately *Spirulina* and *Chlorella*, of various regions of origin, are widely available, not only in pharmacies but also in organic product stores, grocery shops, and supermarkets, as well as through online sales. Considering the increasing popularity of microalgal food products in Poland and recently reported cases of adverse events following the concomitant consumption of certain *Spirulina* and *Chlorella* formulas (Rzymski et al. 2015), it appeared important to further assess the frequency of side effects, consumer satisfaction level, and potential beneficial results associated with the use of such supplements.

## Materials and methods

### Participants and survey

To explore consumers’ behaviours towards the use of microalgal supplements and evaluate their experience with these products, an online survey was conducted. As previously indicated, research based on online questionnaire creates opportunity to collect data nationwide and to reach specific and difficult-to-access groups of individuals (Wright 2005; Rzymski and Królczyk 2016).

The present study employed an anonymous, structured, online questionnaire that concerned:

(i) Consumer behaviours regarding types and forms (pills, tablets, and powder) of microalgal supplements, dosages in which they were taken, and whether a medical doctor or pharmacist was consulted prior to consumption
(ii) Consumer intention(s) behind the use of microalgal supplements
(iii) Main sources from which customers learned about the bioactivity of microalgal supplements
(iv) The frequency of side effects following the use of microalgal supplements as observed by consumers
(v) Consumer satisfaction with the supplement they used and whether they would recommend them to others
(vi) Health-beneficial effects of microalgal supplement use as observed by participants

The demographic characteristics of surveyed individuals were also assessed and included the following: age, gender, economical status, education, type of diet and body mass index (BMI; calculated from weight and height reported in the questionnaire), medical history, and concomitant use of pharmaceutical drugs during the supplementation period.

A questionnaire was made available online for a period of 1 year (June 2015–July 2016). To reach the greatest number of consumers, invitations to complete the questionnaire were frequently made through social media, web portals, and message boards and were distributed through academia and scientific societies in Poland.

### Statistical analyses

The statistical analyses were performed using Statistica v.10.0 (StatSoft, USA). Pearson’s chi-squared test was used to compare the frequencies of the answers among the different groups. Because age and BMI of individuals did not meet the assumption of Gaussian distribution (tested with the Shapiro–Wilk method), the non-parametric Mann–Whitney *U* test was applied to evaluate the effect of these variables on given answers. *P* value < 0.05 was considered as statistically significant.

## Results

### Demographic characteristics

The demographic characteristics of the participants are presented in Table 1. A total of 150 Polish individuals who used microalgal food supplements in the past were considered in this study. The participants had mean age of 35 years, were mostly female, had normal BMI, had tertiary education, and had moderate economic status. Although most individuals were on an omnivorous diet, relatively high incidence of vegetarians (lacto-ovo-vegetarians and vegans) representing 42.7% was noted (Table 1). Kidney failure was declared by 2.7% (*n* = 4) of the participants and hypothyroidism and Hashimoto’s thyroiditis by 3.3% (*n* = 5), each. All individuals from the latter two groups used levothyroxine sodium. Other concomitant drug intakes included the following: lithium (*n* = 1) and perindopril (*n* = 1) with *Chlorella*, betahistine (*n* = 1), and diosmin (*n* = 1) with *Spirulina*.

**Table 1:** Demographical characteristics of microalgal supplement consumers enrolled in the study

Sample group	*Spirulina* (*n* = 85)	*Chlorella* (*n* = 45)	*Aphanizomenon* (*n* = 20)	Total (*n* = 150)
Age (years) (mean ± SD)	34.0 *±* 12.7	35.8 ± 8.3	34.1 ± 5.9	34.6 ± 10.8
Gender				
Female (*n*/%)	65/76.5	37/82.2	12/60.0	114/76.0
Male (*n*/%)	20/23.5	8/17.7	8/40.0	36/24.0
BMI				
Mean ± SD	22.8 ± 4.1	22.5 ± 3.3	22.3 ± 3.5	22.6 ± 3.8
Diet				
Omnivore (*n*/%)	55/64.7	24/53.3	7/35.0	86/57.3
Lacto-ovo-vegetarian (*n*/%)	19/22.4	9/20.0	6/30.0	34/22.7
Vegan (*n*/%)	11/12.9	12/26.7	7/35.0	30/20.0
Education				
Secondary (*n*/%)	27/31.8	13/28.9	6/30.0	46/30.7
Tertiary (*n*/%)	58/68.2	32/71.1	14/70	104/69.3
Economical status				
Low (*n*/%)	5/5.9	4/8.9	0/0.0	9/6.0
Moderate (*n*/%)	65/76.5	32/71.1	14/70.0	111/74.0
High (*n*/%)	15/17.6	9/20.0	6/30.0	30/20.0
Medical history				
Kidney failure (*n*/%)	3/3.5	1/2.2	0/0.0	4/2.7
Hypothyroidism (*n*/%)	2/2.4	2/4.4	1/5.0	5/3.3
Hashimoto’s thyroiditis (*n*/%)	3/3.5	2/4.4	0/0.0	5/3.4

*SD* standard deviation

Most of the study participants used *Spirulina* supplements (*n* = 85; 56.7%); *Chlorella* was used by 30.0% (*n* = 45) whereas formulas based on *Aphanizomenon* by 13.3% (*n* = 20). Tablets were the most popular form in which these products were used (65.3%), followed by powder (24.7%) and capsules (10.0%). The majority of surveyed individuals (74.6%) declared that they had been using supplements for no longer than 3 months; 13.3% had been using them for up to half a year, 4.0% up to 1 year, and 8.0% for longer.

### Consumer behaviour

**Fig. 1:**
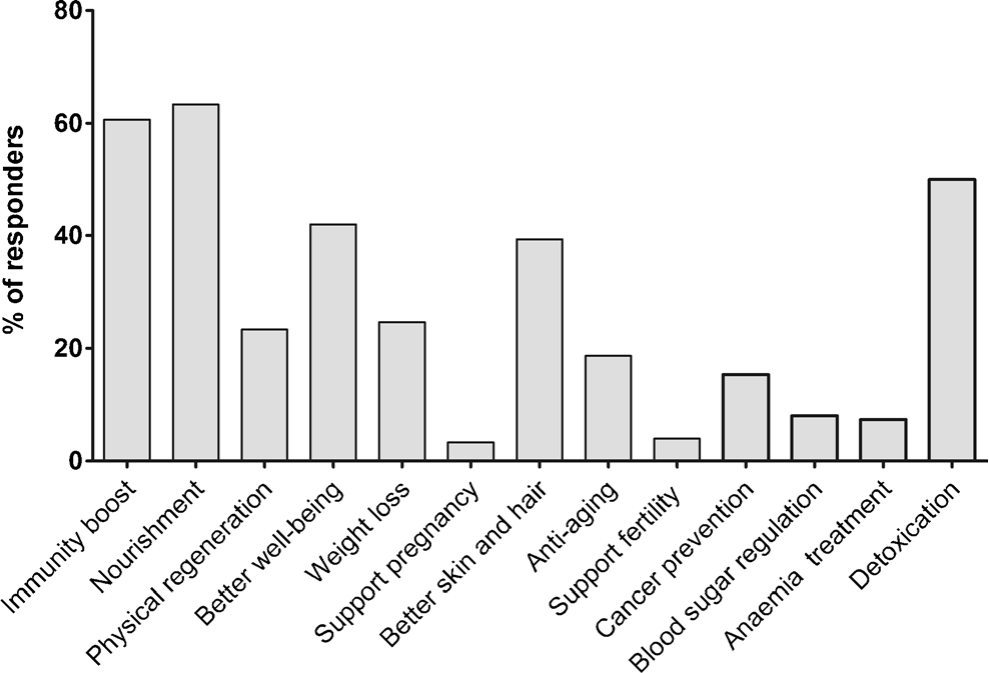
Reasons behind the use of microalgal food supplements in surveyed consumers (*n* = 150)

As declared by the surveyed individuals, microalgal supplements were used for different purposes among which the most popular were the following: supporting the immune function (60.7%), providing nutrients (63.3%), and detoxication (50.0%). A relatively significant number of individuals consumed these products to improve general well-being (42.0%) and skin and hair quality (39.3%), to support weight loss (24.7%), and for physical regeneration (23.3%) (Fig. 1). The reasons given for using supplements did not differ by dietary group apart from nourishment being indicated more often by lacto-ovo-vegetarians (76.5%) and vegans (76.6%) than omnivores (53.5%) (*P* < 0.05).

**Fig. 2:**
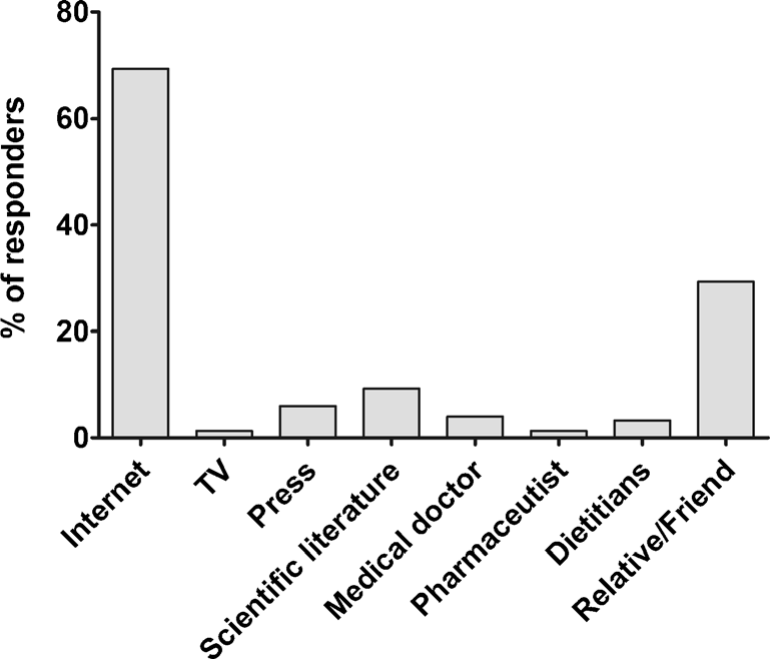
Sources of information on the bioactivity of microalgal supplements among the surveyed consumers (*n* = 150)

The Internet was identified by the vast majority of the studied group (69.3%) to be the main source of information on the bioactivity of microalgal supplements. Medical doctors (4.0%), dietitians (3.3%), and pharmaceutics (1.3%) were one of the least important sources in this regard (Fig. 2). Moreover, most of the participants (88.7%) did not consult their supplementation with either a medical doctor or pharmacist, and none of the *Aphanizomenon* users did.

Generally, 46.0% of the participants stated that they used the supplement at the dosage recommended on the label, 28.0% consumed them at higher or lower doses (14.0% higher, 14.0% lower), while 26.0% were, in turn, unaware of their exact daily intake. The declared daily doses ranged from 0.25 g (*Spirulina*) up to as much as 32 g (*Aphanizomenon*) with an average intake around 4 g per day (Table 2).

**Table 2:** Daily intake doses [g] of microalgal-based food supplements as declared by study participants (*n* = 115). Some individuals (*n* = 35) were unaware of the dose at which the supplement was taken. The recommended daily intakes usually fall below 10 g

	*Spirulina* (*n* = 65)	*Chlorella* (*n* = 39)	*Aphanizomenon* (*n* = 11)	Total (*n* = 115)
Mean ± SD	3.7 ± 3.0	4.1 ± 3.2	6.8 ± 9.3	4.2 ± 4.1
Median	3.0	3.0	3.0	3.0
Min–max	0.25–16.0	1.0–15.0	2.0–32.0	0.25–32.0

### Adverse effects

Overall, the occurrence of self-reported adverse effects in the studied group was 28.7% (*n* = 43) but differed between particular supplement groups (*P* < 0.05). The lowest frequency was observed for *Chlorella* users—17.8% (*n* = 8) with 15 different effects reported (Table 3). In this group, most of the affected individuals (62.5%, *n* = 5) reported only one effect at a time. Greater number of simultaneously occurring adverse events were reported rarely by *Chlorella* users (two, seven, or eight effects recorded by 2.2% (*n* = 1) consumers, each). A slightly higher rate of adverse events, 22.4% (*n* = 19), was observed for *Spirulina* consumers—a total of 21 effects were reported (Table 3). Similar to *Chlorella* users, most of those affected reported one effect at a time (47.4%, *n* = 10) but some observed two (15.8%, *n* = 3), three (5.3%, *n* = 1), four (5.3%, *n* = 1), five (10.5%, *n* = 2), six (10.5%, *n* = 2), and even seven (5.3%, *n* = 1) simultaneously occurring effects. Diarrhoea was the most common effect in groups of *Spirulina* and *Chlorella* users. Up to 80% (*n* = 16) of individuals consuming *Aphanizomenon*-based supplements reported at least one side effect. From 16 events observed by these consumers, the greatest frequency was noted for diarrhoea, nausea, abdominal pain, and general weakness (Table 3). Most of those affected reported four (25.0%, *n* = 4) and five (25.0%, *n* = 4) simultaneously occurring effects. One or two effects at a time were observed by 12.5% (*n* = 2) individuals, each; six or 12 by 6.25% (*n* = 1), each. Additionally, 6.7% (*n* = 3) and 8.2% (*n* = 7) of *Chlorella* and *Spirulina* consumers, respectively, reported a metallic taste in the mouth following their consumption of the supplement; none noted it after the use of *Aphanizomenon* supplement. Individuals reporting side effects declared that they had been using microalgal supplement for no longer than 1 month.

**Table 3:** Adverse events self-reported by microalgal consumers (*n* = 150)

	*Spirulina* (*n* = 85)	*Chlorella* (*n* = 45)	*Aphanizomenon* (*n* = 20)
	% (*n*)		
Abdominal pain	7.0 (6)	2.2 (1)	40.0 (8)
Appetite loss	1.2 (1)	–	15.0 (3)
Bone pain	2.4 (2)	2.2 (1)	–
Constipation	4.7 (4)	6.6 (3)	5.0 (1)
Diarrhoea	12.9 (11)	6.6 (3)	70.0 (14)
Dizziness	2.4 (2)	2.2 (1)	10.0 (2)
Dysuria	1.2 (1)	–	–
Fever	2.4 (2)	2.2 (1)	10.0 (2)
General weakness	7.0 (6)	4.4 (2)	40.0 (8)
Headache	7.0 (6)	4.4 (2)	5.0 (1)
Hearing problems	1.2 (1)	2.2 (1)	–
Hypertension	2.4 (2)	–	–
Hypotension	1.2 (1)	–	10.0 (2)
Insomnia	1.2 (1)	2.2 (1)	15.0 (3)
Muscles pain	2.4 (2)	4.4 (2)	5.0 (1)
Nausea	10.6 (9)	2.2 (1)	50.0 (10)
Skin itching	3.5 (3)	2.2 (1)	10.0 (2)
Skin rash	7.0 (6)	4.4 (2)	10.0 (2)
Tachykardia	1.2 (1)	–	5.0 (1)
Vision problems	1.2 (1)	2.2 (1)	–
Vomiting	2.4 (2)	0.0 (0)	20.0 (4)

Consumer age, BMI, and gender had no significant effect on the occurrence of any of the adverse events in any supplement group (*P* > 0.05 in all cases). The frequency of self-reported adverse events was also unaffected by the supplement form (tablets, pills, or powder) and consumed doses, regardless of the supplement group (*P* > 0.05 in all cases). Within the *Spirulina* group, adverse effects were significantly more often encountered (*P* < 0.05) by vegans (63.6%) than lacto-ovo-vegetarians (5.3%) and omnivores (0.0%). Importantly, all individuals (*n* = 3) suffering from kidney failure and consuming *Spirulina* products reported side effects: diarrhoea (*n* = 2) and headache (*n* = 1). The only identified *Chlorella* consumer with kidney failure reported a variety of adverse events including headache, muscle pain, fever, insomnia, hearing and vision problems, and general weakness. No significantly increased total frequency of side effects was observed for Hashimoto’s thyroiditis subjects (only a single individual reported nausea after using *Chlorella*) compared to healthy subjects (*P* > 0.05). An increased rate of these effects was, however, found for hypothyroidism; four of five (80%) individuals noted them. In this group, one person reported headaches, dizziness, nausea, bone pain, skin rash, and general weakness following the use of *Spirulina*, while a second individual reported diarrhoea. Constipation, abdominal pain, headaches, dizziness, bone and muscle pain, skin rash, and general weakness were reported by one person after using *Chlorella*. Finally, one hypothyroic subject consuming *Aphanizomenon* supplement reported diarrhoea, abdominal pain, nausea, hypotension, and skin rash. The patient consuming *Chlorella* concomitantly with lithium reported diarrhoea and nausea. No side effects were reported by patients using *Spirulina* with diosmin (*n* = 1) or betahistine (*n* = 1) and *Chlorella* with perindopril (*n* = 1). Adverse effects were encountered significantly less often by individuals consulting a medical doctor or pharmacist about the microalgal supplementation than in the group of those who did not consult one (5.9 vs 31.6%, *P* < 0.05).

### Beneficial effects and consumer satisfaction

A number of beneficial effects, with varying frequencies, were reported by the group using *Spirulina*- (total of 16 effects) and *Chlorella*-based (total of 13 effects) supplements (Table 4). The most often reported positive outcomes from the former group included higher vitality, immunity boost, improved general well-being, and better skin quality. Most of the *Spirulina* consumers (54.1%) were satisfied with the supplement (12.9% were dissatisfied and 32.9% could not unambiguously decide), and most of them (58.8%) would recommend its use (11.8% would not and 29.4% could not unambiguously decide). The *Chlorella* users declared better skin quality and general well-being, higher vitality, and improvement with defecation with the greatest frequencies. Similar to *Spirulina*, most of the consumers (55.6%) were satisfied with their consumed *Chlorella* supplement (13.3% were dissatisfied and 31.1% could not unambiguously decide) whereas 57.8% would recommend its use (13.3% would not and 28.9% could not unambiguously decide). In turn, individuals using *Aphanizomenon*-based supplements did not observe any beneficial effects of supplementation, none of them were satisfied with these products (85.0% were not satisfied and 15.0% could not unambiguously decide), and 80.0% would not recommend them to others (20.0% could not unambiguously decide).

**Table 4:** Health benefits of microalgal supplement consumption as self-reported by the users. None were reported for consumers of *Aphanizomenon*-based products (*n* = 20)

	*Spirulina* (*n* = 85)	*Chlorella* (*n* = 45)
	% (*n*)	
Allergy relief	4.7 (4)	–
Asthma relief	3.5 (3)	–
Better general well-being	8.2 (7)	22.2 (1)
Better hair	8.2 (7)	4.4 (2)
Better skin	9.4 (8)	22.2 (10)
Cheilosis relief	–	2.2 (1)
Feeling of satiety	5.9 (5)	4.4 (2)
Headache relief	–	2.2 (1)
Higher vitality	17.6 (15)	20.0 (9)
Improved memory and focus	–	2.2 (1)
Improved B_12_ vitamin status	–	2.2 (1)
Improved iron status	1.2 (1)	4.4 (2)
Improvement with defecation	1.2 (1)	11.1 (5)
Increased immunity	14.1 (12)	2.2 (1)
Insomnia reduction	1.2 (1)	–
Libido increase	1.2 (1)	–
Normalized pressure	1.2 (1)	–
Rectal itching relief	1.2 (1)	–
Stopping cold	2.4 (2)	–
Weight reduction	2.4 (2)	4.4 (2)

## Discussion

The present study has provided the insight into the world of microalgal consumers. It identifies their basic demographic characteristic, motivations and other behaviours behind the use of microalgal supplements (with significance for the industry), self-reported beneficial effects of supplementation (relevant for research on the bioactivity of microalgae), the daily doses at which these supplements are taken, and the frequency of adverse events following the use of particular types of microalgal formulas (important for product safety evaluation). However, further investigations should be conducted to ensure that these microalgal supplements are the cause of the adverse effects as well as benefits.

Microalgal supplements are classified and regulated as foodstuffs although their labelling in the EU and other world regions requires certain particulars among which are the following: (i) the portion of the product recommended for daily consumption, (ii) a warning not to exceed the stated recommended daily dose, and (iii) a statement to the effect that food supplements should not be used as a substitute for a varied diet (Directive 2002/46/EC). It is highly advisable that consumers comply with and not exceed the recommended doses. All microalgae contain high protein and chlorophyll content which at excess can cause gastrointestinal issues such as diarrhoea, nausea, or cramps (Garlick 2004) and high levels of phosphorus of which excessive intake can have deleterious effects on the kidneys (Uribarri and Calvo 2013). Moreover, all microalgal supplements represent a rich source of iron; overloading of which has also been associated with gastrointestinal distress (Frykman et al. 1994) as well as the generation of reactive oxygen species through Fenton and Haber–Weiss reactions and subsequent oxidative stress (Galaris and Pantopoulos 2008). Elevated intake of manganese, which is also present at high concentrations in microalgal supplements, can, in turn, exhibit neurotoxic effects (Neal and Guilarte 2013). Therefore, it is recommended that potential consumers consult the use of these products with a medical doctor, a pharmacist, or a professional dietitian and, preferably, precede the supplementation with biochemical tests (e.g. iron status and haemoglobin concentration). It is beyond any doubt that the nutritional value of microalgae is high enough to be beneficial (Khan et al. 2005; Buono et al. 2014; Merchant et al. 2015), but their safe use has to be individually justified by evidence-based means rather than intuition. Further, the immunomodulatory effects of these supplements can be beneficial for some individuals (Selmi et al. 2011; Kwak et al. 2012) but are rather less desirable in patients suffering from autoimmune diseases such as pemphigus vulgaris (Lee and Werth 2004). The excessive or inappropriate use of any food supplement can be harmful, thus consulting a health care provider prior to use can only be beneficial (Marcus 2016).

One of the most striking findings of the present study is the high incidence of side effects reported by consumers of *Aphanizomenon* supplements. Besides gastrointestinal symptoms such as diarrhoea, nausea, or abdominal pain, which can generally be classified as a low/moderate health threat, skin rash and itching were also reported, indicating inducement of generalized allergic reactions following the use of a supplement. Such allergenicity of detoxified *Aphanizomenon flos-aquae* has already been shown in a human study employing skin-prick testing, at a frequency of 12% (Bernstein et al. 2011). In addition, commercial powder based on biomass of *A. flos-aquae* harvested from Klamath Lake has been toxic to human dermal fibroblasts (Niccolai et al. 2016). Moreover, it is worth highlighting that *Aphanizomenon*-based supplements raise numerous controversies after hepatotoxic cyclic peptides, microcystins, and neurotoxic alkaloid anatoxin-a have been identified in some of these products (Rellán et al. 2009). Microcystins have never been identified in *Aphanizomenon* sp., and the presence of these compounds most likely results from the co-occurrence of toxic species (e.g. *M. aeruginosa*) in the environment in which the target species is harvested from (Carmichael et al. 2000; Gilroy et al. 2000). However, the biosynthesis of anatoxin-a is known to occur in the former ‘*Aphanizomenon*’ genus, e.g. in *Cuspidothrix* (formerly *Aphanizomenon*) *issatschenkoi*, which may be difficult to distinguish from *A. flos-aquae* by means of morphological features only (Rapala et al. 1993; Ballot et al. 2010). Further, some strains of *A. flos-aquae* have been demonstrated to produce cylindrospermopsin alkaloid (Preussel et al. 2006) and paralytic shellfish poisoning toxins (Ferreira et al. 2001). Identification of these strains requires analytical studies and/or molecular screening to exclude the possibility of harvesting toxic forms, yet such an approach remains more a subject of scientific investigation than part of a thorough monitoring programme. Nevertheless, some promising effects of *Aphanizomenon* supplementation such as a decrease in blood glucose and glycated haemoglobin levels in patients with type 2 diabetes (Sanaei et al. 2015) and improved B_12_ status in vegan subjects have been demonstrated (Baroni et al. 2009) but they may not overcome potential health threats. Altogether, it appears that these microalgal products may require particularly strict monitoring in regard to toxin content to ensure consumer safety.

The frequency of side effects following the use of *Spirulina* and *Chlorella* was significantly lower than that for *Aphanizomenon* supplements, although it still accounted for 18–22% of their consumers. The most frequently reported events were non-serious and included diarrhoea, nausea, dizziness, and headaches. These symptoms, along with vomiting and abdominal cramps, were also the most common side effects identified in *Spirulina* users as indicated by a collective report of existing data from FDA MedWatch, Health Canada, Australian Adverse Event Reports, and Uppsala Monitoring Centre (World Health Organization Collaborating Centre for International Drug Monitoring) (Marles et al. 2011). Additionally, more serious events have been recorded including liver damage, transient ischemic attack, heart palpitations, or severe parkinsonian syndrome (Marles et al. 2011). Prior to the present study, there was no compiled data on adverse symptoms associated with *Chlorella use*; the only events that had been reported included a unique case of recurrent psychosis (Selvaraj et al. 2013), five cases of photosensitization (Jitsukawa et al. 1984), a single event of asthma (Ng et al. 1994), and skin rash and itching, nausea, vomiting, and muscle and bone pain following the concomitant use of low doses of *Spirulina* and *Chlorella* (Rzymski et al. 2015). It should be stressed that *Arthrospira* sp. and *Chlorella* sp. are not known to produce any known toxic metabolites and are generally considered per se as safe for human consumption (Hutadilok-Towatana et al. 2010; Yang et al. 2011). It is therefore plausible that the adverse events are mostly due to product contamination—avoidable by maintaining quality assurance and monitoring (Grobbelaar 2003; Rzymski et al. 2015; Wells et al. 2016).

Little is known about the interactions between microalgal supplements and medical drugs. It can only be hypothesized that due to the immunostimulatory activity of microalgal components, their intake may interfere with immunosuppressive drugs although no clinical evidence exists in this matter. The present study demonstrated a wide range of side effects of *Spirulina*, *Chlorella*, and *Aphanizomenon* use in individuals suffering from hypothyroidism. This finding is important if one considers that hypothyroidism is a common disorder and its prevalence is likely to increase globally (Gaitonde et al. 2012). Conversely, the frequency of side effects was very low in autoimmune Hashimoto’s thyroiditis although all individuals, similar to the hypothyroidism group, concomitantly used levothyroxine sodium. Therefore, the observed effects cannot be solely linked to the interaction between thyroid hormone and bioactive ingredients of microalgal supplements. Further research is necessary to elucidate the potential risks arising from the use of these products by hypothyroic subjects.

It came as no surprise that the Internet was the most important source of information on microalgal food supplements, as its influential role in diet and food promotion has already been well documented (Pettigrew et al. 2013; Rzymski and Królczyk 2016). The benefits of microalgae as food are widely promoted through different message boards, social media websites, and diet-dedicated portals. It is highly important for the microalgal industry to communicate information on the bioactivity of supplements to the general public in an accurate and evidence-based manner, avoiding misunderstandings, over-interpretations, and hypes. For example, Heussner et al. (2012) reported that some microalgal supplements were specifically marketed as a supportive treatment for patients with attention-deficit hyperactivity disorder, although to date, no study has proven such a use to be either effective or harmless for such subjects.

In the present study, most of the consumers admitted to use microalgal supplements to support their immune function and provide nutrients. Both reasons are supported by existing experimental data and clinical trials (Park et al. 2008; Kwak et al. 2012; Wells et al. 2016); thus, they can be considered reasonable. However, half of the respondents declared that their intention had been to use these products to remove toxins (e.g. heavy metals) from the body, a claim which is not evidenced by a single scientific study but promoted by unreliable Internet sources. There is also no data demonstrating that microalgal supplements may support human fertility. Their use in pregnancy was, in turn, tested only for *Chlorella* and revealed no adverse effects on mother or offspring but significantly reduced leg swelling in the third trimester, while risk of anaemia and proteinuria was noted (Nakano et al. 2010). Although promising results were demonstrated in in vivo rodent experimental models for *Spirulina* supplementation in pregnancy (Pankaj 2015), one case study linked severe neonatal hypercalcaemia following maternal use of *Spirulina* from the fourth month of pregnancy (Moulis et al. 2012). Further human studies are required to assess the safety of these products for pregnant women and their offspring. The beneficial effect of supplementation on hair and skin quality is also not directly evidenced but hypothetically plausible if one considers the nutritional value of these supplements, particularly high content of fatty acids and vitamins. In the present study, a relatively high percentage of those participants declared that *Chlorella* and *Spirulina* intake actually improved the quality of their hair and skin; thus, further studies should be conducted to clinically validate such a function of microalgae.

Although the present study revealed some valuable information on microalgal supplements, there are some limitations and interpretation of the results should be approached with caution. The anonymous and online character of the survey excluded the possibility of verifying the data, particularly that concerning the occurrence of adverse and beneficial effects, on the clinical level. Thus, it is plausible that some data were misinterpreted by the respondents or resulted from a placebo-like effect. Moreover, the study did not evaluate the quality of particular supplements that participants consumed, so one cannot unambiguously conclude whether the adverse events resulted from the interactions of natural microalgal constituents, product contamination (e.g. toxic metals, cyanotoxins from culture-contaminated species, and bacterial contamination), or individual susceptibility. The study is also limited to one country although it aimed to reach as many Polish consumers of microalgal supplements as possible. Their total number of 150 is relatively low for a country with a population of over 38 million. On the other hand, it can be considered satisfying if one considers specificity of the studied group and that all microalgal products available in Poland are imported (lack of internal production limits the popularity of these products).

In summary, the present study highlights that microalgal supplements are consumed mostly for their nutritional, immune-boosting, and allegedly detoxifying activities, at different, sometimes higher than recommended (up to 32 g daily), doses. Their use is rarely discussed with specialists; it is rather the Internet that provides the most important source of information on these products. The side effects of supplementation are mostly of mild character and are faced more frequently by individuals with kidney failure and hypothyroidism and less often by those who consult a medical doctor/pharmacist about their use. Autoimmune Hashimoto’s thyroiditis was preliminarily not found to be associated with increased frequency of side effects of microalgal supplement consumption—the observation requiring further testing before final conclusion can be drawn. The high frequency of adverse events and dissatisfaction with product use in the group consuming *Aphanizomenon*-based products raise questions concerning the safety of these supplements. The study highlights the need to monitor microalgal supplements for contamination and underlines that labelling these products with warnings of potential side effects and recommendation to consult a qualified health specialist (e.g. medical doctor, dietician, and pharmacist) prior to use would be of potential benefit for consumer safety.
